# Coronavirus Disease 2019 in Patients With Prior Ischemic Stroke

**DOI:** 10.7759/cureus.10231

**Published:** 2020-09-03

**Authors:** Krishna Nalleballe, Suman Siddamreddy, Sen Sheng, Vasuki Dandu, Narenraj Arulprakash, Sukanthi Kovvuru, Mudassar Kamran, Madhu Jasti, Sanjeeva Onteddu

**Affiliations:** 1 Neurology, University of Arkansas for Medical Sciences, Little Rock, USA; 2 Internal Medicine, Baptist Health Medical Center, Little Rock, USA; 3 Neurology, Baptist Health Medical Center, Little Rock, USA; 4 Radiology, University of Arkansas for Medical Sciences, Little Rock, USA; 5 Neurology, University of Maryland, Glen Burnie, USA

**Keywords:** covid 19, stroke

## Abstract

Background: It is uncertain if patients with prior ischemic stroke are vulnerable to coronavirus disease 2019 (COVID-19) and its complications.

Methods: We used TriNetX, a global health collaborative clinical research platform with a large global COVID-19 database. COVID-19 infection was identified with a positive lab value for severe acute respiratory syndrome coronavirus 2 (SARS-CoV-2) and related ribonucleic acid (RNA).

Findings: A total of 604,258 patients with history of ischemic stroke were identified, of which 891 patients (study cohort) were diagnosed with COVID-19. A control cohort with 32,136 patients diagnosed with COVID-19 after January 20^th^ 2020 without a history of ischemic stroke were identified. A comparison between study cohort and control cohort showed patients with prior history of stroke (study cohort) were older (69.5 vs 47.8; p<0.0001) and had more comorbidities contributing to worse clinical outcomes. After propensity matching for demographic variables and comorbidities, only rate of hospitalization (287 vs 231; p=0.0035) and need for critical care services (85 vs 55; p=0.0082) remained statistically significant while intubation (51 vs 43; p=0.39) and death (119 vs 115; p=0.77) showed trends towards worse outcomes but were not statistically significant.

Interpretation: Patients with history of ischemic stroke tend to be significantly older with several comorbid conditions contributing to worse clinical outcomes after COVID-19, which makes them a vulnerable population.

## Introduction

A novel coronavirus, severe acute respiratory syndrome coronavirus 2 (SARS-CoV-2), emerged in December 2019 and soon led to a worldwide pandemic, coronavirus disease 2019 (COVID-19) [1]. COVID-19 has been shown to cause several neurological manifestations including ischemic strokes [2-4]. Most of the predictions about risk factors, clinical characteristics, and pathophysiology are based on our experience with previous coronaviruses, i.e. severe acute respiratory syndrome coronavirus (SARS-CoV) and Middle East respiratory syndrome coronavirus (MERS-CoV). Stroke is one of the chronic diseases with very high morbidity and mortality causing a huge financial burden to the healthcare economy [5]. While there are many studies looking at risk factors for severe COVID-19 and its complications, ‘history of ischemic stroke’ as a risk factor contributing to the severity of COVID-19 has not been studied in a large population [6]. The purpose of this study is to explore if patients with prior ischemic stroke are a vulnerable population for worse clinical outcomes in patients with COVID-19.

## Materials and methods

Data source

De-identified patient information data for COVID-19 and history of ischemic stroke were extracted from TriNetX (www.trinetx.com), which is a global health collaborative clinical research platform collecting real-time electronic medical records data from a network of health care organizations (HCOs). “COVID-19 Research Network” in TriNetX is a large COVID-19 database, which is also being used by the Food & Drug Administration (FDA) Sentinel Operations Center at the Harvard Pilgrim Health Care Institute to monitor priority drugs used for the care of the hospitalized COVID-19 patients [7]. 

Queries were made through TriNetX using an internet browser and accessing its real-time features. TriNetX does not allow data downloads or individual patient data for review. However, the TriNetX platform allows analysis in the form of queries. The TriNetX platform has previously been described in detail in several similar studies on COVID-19 that used this platform [3,8,9,10,11]. At University of Arkansas for Medical Sciences, the data from TriNetX is managed by the Arkansas Clinical Data Repository (AR-CDR) and maintained by the Department of Biomedical Informatics. 

Study protocol

The University of Arkansas Institutional Review Board (IRB) deemed this study to be ‘not human subject research’ (global de-identified COVID-19 Research Network data designated for research use), and gave it an IRB exempt status. 

Data were accessed on July 20, 2020 on TriNetX COVID-19 Research Network. Using ICD-10 codes, individuals ≥ 18 years of age with a pre-existing ischemic stroke (I63) were identified. Among them, we then examined patients with a confirmed lab-positive reverse transcription polymerase chain reaction (RT-PCR) for COVID-19 (study cohort). For comparison, a control cohort with 32,136 patients diagnosed with COVID-19 after January 20, 2020 without a history of ischemic stroke were identified. Between the two cohorts, hospitalizations, critical care services, intubation, and mortality data within one month from COVID-19 diagnosis were studied. Additionally, baseline demographics data and clinical outcomes in these cohorts were examined. Statistical analysis was performed using the TriNetX analytics function. Descriptive statistics are reported as number of observations, percentage, or mean ± standard deviation as applicable. One-to-one propensity score matching was done for baseline characteristics and comorbid conditions. The p-value for variables is generated from a t-test. For the outcomes however, a z-test is used for the p-value.

## Results

There were 604,258 patients with prior history of ischemic stroke, of which 891 (0.15%) patients developed COVID-19 (study cohort). Among them 451 (50.6%) were females; 409 (45.9%) Caucasians, 278 (31.2%) African Americans and 32 (3.6%) Asians. Ethnicity and comorbid conditions are detailed in Table 1. The control cohort included 32,136 patients. Baseline characteristics, gender, race, comorbid conditions of this cohort are also detailed in Table 1. 

**Table 1:** Table 1. Characteristics and outcomes of patients with COVID-19 Baseline demographics, comorbidities and outcomes for Study and Control cohort. Study Cohort: Patients with COVID-19 and history of ischemic stroke. Control Cohort: Patients with COVID-19 without history of ischemic stroke. [NA-Not applicable]

	Study Cohort	Control Cohort	p-value
Number of patients	891	32,136	NA
Age (years)	69.5 ± 14.5	47.8 ± 18.9	< 0.0001
Woman	451(50.6%)	16,956(52.7%)	0.21
Race			
White	409(45.9%)	12,220(38.0%)	< 0.0001
African American	278(31.2%)	6,177(19.2%)	< 0.0001
Asian	32(3.6%)	641(1.9%)	0.0009
Hispanic (ethnicity)	68(7.6%)	4,564 (14.2%)	< 0.0001
Co morbid conditions			
Hypertension	734(82.4%)	7,489(23.3%)	< 0.0001
Diabetes mellitus	426(47.8%)	3,986(12.4 %)	< 0.0001
Ischemic heart disease	394(44.2%)	2,102(6.5%)	< 0.0001
Chronic kidney disease	297(33.3%)	1,617(5.0%)	< 0.0001
Heart failure	303(34.0%)	1,453(4.5%)	< 0.0001
Atrial fibrillation & flutter	241(27.1%)	1,136(3.6%)	< 0.0001
Obstructive pulmonary disease	188(21.1%)	1,125(3.5%)	< 0.0001
Overweight & obesity	297(33.3%)	4,603(14.3%)	< 0.0001
Nicotine dependence	169(18.9%)	2,039(6.3%)	< 0.0001
Liver Disease	79(8.9%)	1,251(3.9%)	< 0.0001
Clinical outcome			
Hospitalization	289(32.4%)	3,746(11.7%)	< 0.0001
Critical care services	85(9.5%)	711(2.2%)	< 0.0001
Intubation	51(5.7%)	642(1.9%)	< 0.0001
Death	119(13.4%)	1,143(3.6%)	< 0.0001

Among these two cohorts, patients in the study cohort were older (69.5 years vs 47.8 years; p<0.0001), had significantly higher prevalence of hypertension, diabetes mellitus, ischemic heart disease, chronic kidney disease, heart failure, atrial fibrillation and flutter, obstructive pulmonary disease, overweight and obesity, liver disease, and nicotine dependence (p<0.0001, Table 1). When comparing for COVID-19 outcomes, unmatched analysis showed statistically higher rates of hospitalization (32.4% vs 11.7%, p<0.0001), need for critical care services (9.5% vs 2.2%, p<0.0001), intubation (5.7% vs 1.9%, p<0.0001) and death (13.4% vs 3.6%, p<0.0001) (Table 1) among the study cohort. After propensity score matching for demographics and comorbidities, each group had 888 patients. Rate of hospitalization (32.3% vs 26.0%, p=0.0035) and need for critical care services (9.5% vs 6.2%, p=0.0082) was higher in the study cohort and was statistically significant. While intubation (5.7% vs 4.8%, p=0.39) and death (13.4% vs 12.9%, p=0.77) showed trends towards worse outcomes they were not statistically significant (Table 2). 

**Table 2:** Characteristics and outcomes of patients with COVID-19 after propensity matching Baseline demographics, comorbidities and outcomes for Study and Control cohort. Study Cohort: Patients with COVID-19 and history of ischemic stroke. Control Cohort: Patients with COVID-19 without history of ischemic stroke. Composite end point includes hospitalizations, critical care services, intubations and death. [NA-Not applicable]

	Study Cohort	Control Cohort	p-value
Number of patients	888	888	NA
Age (years)	69.5 ± 14.5	70 ± 14.9	0.48
Woman	450(50.7%)	433(48.8%)	0.42
Race			
White	409(46.1%)	422(47.5%)	0.54
African American	277(31.2%)	258(29.1%)	0.33
Asian	30(3.4%)	31(3.5%)	0.89
Hispanic (ethnicity)	68(7.7%)	67(7.6%)	0.93
Co morbid conditions			
Hypertension	731(82.3%)	751(84.6%)	0.2
Diabetes mellitus	424(47.8%)	423(47.6 %)	0.96
Ischemic heart disease	391(44.0%)	372(41.9%)	0.36
Chronic kidney disease	294(33.1%)	282(31.8%)	0.54
Heart failure	300(33.8%)	278(31.3%)	0.26
Atrial fibrillation & flutter	239(26.9%)	242(27.2%)	0.87
Obstructive pulmonary disease	187(21.1%)	181(20.4%)	0.73
Overweight & obesity	295(33.2%)	298(33.6%)	0.88
Nicotine dependence	168(18.9%)	144(16.2%)	0.13
Liver Disease	79(8.9%)	84(9.5%)	0.68
Clinical outcome			
Hospitalization	287(32.3%)	231(26.0%)	0.0035
Critical care services	85(9.5%)	55(6.2%)	0.0082
Intubation	51(5.7%)	43(4.8%)	0.39
Death	119(13.4%)	115(12.9%)	0.77
Composite end point	198(22.3%)	161(18.1%)	0.0288

A composite endpoint which included hospitalizations, critical care services, intubations and death was significant in patients with prior history of ischemic strokes (22.3% vs 18.1%, p=0.0288).

## Discussion

In this study, we explored if prior ischemic stroke is an independent risk factor for worse clinical outcomes in patients with COVID-19. Identifying vulnerable populations may allow patients and healthcare professionals to take extra precautions to prevent the occurrence of disease as well as its complications, which may eventually help in decreasing the burden on the health care economy.

A history of prior stroke has been shown to be associated with worse outcomes in SARS-CoV and MERS-CoV [12,13]. Even in other respiratory illnesses, like community-acquired pneumonia, stroke has been associated with higher mortality [14]. A recent meta-analysis showed that COVID-19 patients with underlying hypertension, cardiovascular disease, and previous respiratory diseases are at higher risk of having severe disease when compared to patients without those comorbidities [15]. Patients with previous cardiovascular disease have increased risk of death with COVID-19 infection [16]. Pooled analysis of six studies done by Aggarwal et al. showed that there is a 2.5-fold increase in odds of severe COVID-19 infection in patients with previous strokes, but no significant association with mortality; however, the analysis had few studies with small sample size [17]. Similarly, a study by Qin et al. showed that after propensity score matching patients with history of stroke had higher risk of severe events including mortality, critical care admission, intubations, and lower rates of discharge [6]. Patients with history of stroke tend to be older with several comorbidities which likely resulted in severe COVID-19 complications. After propensity matching, we noticed that history of ischemic stroke does not seem to be an independent risk factor for intubations and deaths. 

There are a few limitations of this study, some of them are inherent to large databases. First, findings are based on reported data, and accuracy of reported data could not be verified. Second, we could not assess the duration and severity of prior ischemic stroke.

## Conclusions

Patients with history of prior ischemic stroke tend to be older with several comorbid conditions, which contribute to severe COVID-19 complications and make them a vulnerable population. Future large prospective studies may provide additional insights into this relationship.
